# A solar tower fuel plant for the thermochemical production of kerosene from H_2_O and CO_2_

**DOI:** 10.1016/j.joule.2022.06.012

**Published:** 2022-07-20

**Authors:** Stefan Zoller, Erik Koepf, Dustin Nizamian, Marco Stephan, Adriano Patané, Philipp Haueter, Manuel Romero, José González-Aguilar, Dick Lieftink, Ellart de Wit, Stefan Brendelberger, Andreas Sizmann, Aldo Steinfeld

**Affiliations:** 1 Department of Mechanical and Process Engineering, ETH Zurich, 8092 Zurich, Switzerland; 2 Unit of High-Temperature Processes, IMDEA Energy, 28935 Móstoles, Spain; 3 HyGear Technology and Services B.V., 6827 AV Arnhem, the Netherlands; 4 Institute of Future Fuels, German Aerospace Center (DLR), 51147 Cologne, Germany; 5 Bauhaus Luftfahrt e.V., 82024 Taufkirchen, Germany

**Keywords:** concentrated solar energy, thermochemical, solar fuels, kerosene, solar reactor, redox cycle, water splitting, CO2 splitting, ceria, sustainable aviation fuels.

## Abstract

Developing solar technologies for producing carbon-neutral aviation fuels has become a global energy challenge, but their readiness level has largely been limited to laboratory-scale studies. Here, we report on the experimental demonstration of a fully integrated thermochemical production chain from H_2_O and CO_2_ to kerosene using concentrated solar energy in a solar tower configuration. The co-splitting of H_2_O and CO_2_ was performed via a ceria-based thermochemical redox cycle to produce a tailored mixture of H_2_ and CO (syngas) with full selectivity, which was further processed to kerosene. The 50-kW solar reactor consisted of a cavity-receiver containing a reticulated porous structure directly exposed to a mean solar flux concentration of 2,500 suns. A solar-to-syngas energy conversion efficiency of 4.1% was achieved without applying heat recovery. This solar tower fuel plant was operated with a setup relevant to industrial implementation, setting a technological milestone toward the production of sustainable aviation fuels.

## Highlights

• Entire process chain from H_2_O and CO_2_ to solar kerosene realized in a solar tower
• 50-kW solar reactor demonstrated for ceria-based thermochemical redox splitting
• Consecutive redox cycling produced syngas suitable for FT synthesis
• 4.1% solar-to-syngas energy efficiency achieved without implementing heat recovery

## Context & scale

The aviation sector, which strongly relies on fossil-derived kerosene, is responsible for vast amounts of anthropogenic greenhouse gas emissions. To avoid these emissions, solar energy can be leveraged to efficiently produce sustainable drop-in fuels, e.g., solar--made synthetic kerosene, which is fully compatible with the existing global jet fuel infrastructures for its storage, distribution, and end-use in jet engines. This work advances the technological readiness level of solar fuels production by demonstrating the technical feasibility of the entire sun-to-liquid fuel process chain, from H_2_O and CO_2_ to kerosene, in a pilot-scale solar tower. We evaluate the performance of the solar reactor—the cornerstone technology—based on five primary metrics (namely, reaction selectivity, syngas quality, fuel purity, energy efficiency, and material stability) and experimentally validate its stable operation and full integration in the solar tower fuel plant.

## Graphical abstract

**Figure:**
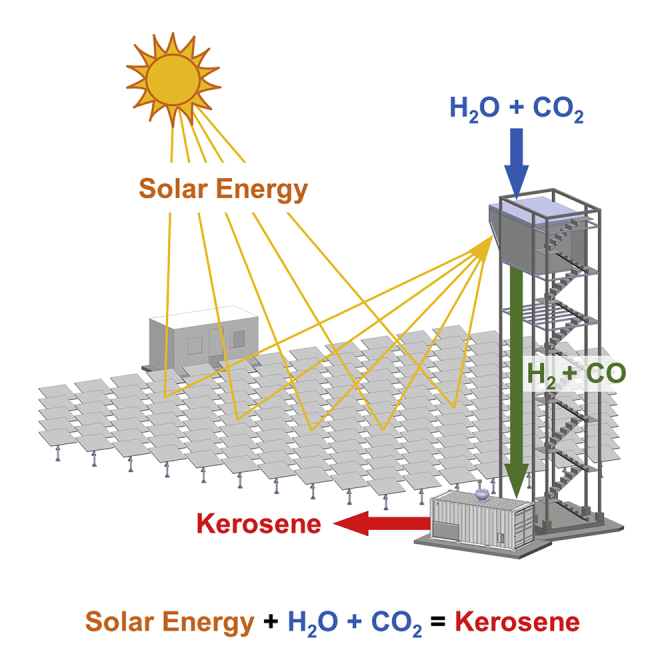

## Teaser

For the first time, the thermochemical production of kerosene using solar energy, water, and CO_2_ is demonstrated in a fully integrated solar tower fuel plant. Solar-made kerosene can replace fossil-derived kerosene and further make use of the existing global jet fuel infrastructures and engines, which are particularly critical for the long-haul aviation sector. This pioneer technological demonstration, performed at a pilot scale relevant to industrial implementation, represents a critical milestone on the path toward the production of sustainable aviation fuels.

## Introduction

For the foreseeable future, kerosene will be indispensable as a jet fuel for long-haul aviation due to its high specific gravimetric energy density and compatibility with the existing global fuel infrastructure. However, approximately 5% of current anthropogenic emissions causing climate change are attributed to global aviation, and this number is expected to increase.^1^ An alternative to conventional kerosene derived from petroleum is kerosene synthesized from syngas—a specific mixture of H_2_ and CO—via the established Fischer-Tropsch (FT) synthesis process. The technological challenge, however, is to produce renewable syngas from H_2_O and CO_2_ using solar energy. The solar-driven thermochemical splitting of H_2_O and CO_2_ via a two-step metal oxide redox cycle can meet this challenge.^2^ Such a process offers a thermodynamically favorable pathway to syngas production because it uses the entire solar spectrum as the source of high-temperature process heat for effecting the thermochemical conversion, and it does so with high reaction rates and potentially high efficiencies.^3^^,^^4^ An additional advantage of the solar redox cycle compared with other solar approaches is its ability to co-split H_2_O and CO_2_ simultaneously or separately and therefore control the quality (both purity and stoichiometry) of the syngas *in situ*, consequently obtaining a tailored mixture of H_2_ and CO suitable for FT synthesis.^5^ This direct approach eliminates the energy penalty associated with additional refinement steps for adjusting the syngas mixture. In contrast, the electrolytic pathway (also called “power-to-X”)^6^ requires the production of substantial excess H_2_ by water electrolysis using solar electricity that is subsequently consumed via the reverse water-gas shift reaction (RWGS reaction: H_2_ + CO_2_ = H_2_O + CO, endothermic by 95.9 kJ/mol above 800°C) to obtain syngas suitable for FT synthesis. As will be shown in this study, the thermochemical approach bypasses the solar electricity generation, the electrolysis, and the RWGS steps, directly producing solar syngas of desired composition for FT synthesis, i.e., three steps are replaced by one.

Ceria (CeO_2_) is currently considered the state-of-the-art redox material because of its rapid redox kinetics and long-term stability.^7^ The two-step thermochemical redox cycle is represented by:

(Equation 1) $$Reduction:\;CeO_{2}\to CeO_{2-\delta }+\frac{\delta }{2}O_{2}$$

(Equation 2a) $$Oxidation:\;CeO_{2-\delta }+\delta H_{2}O\to CeO_{2}+\delta H_{2}$$

(Equation 2b) $$CeO_{2-\delta }+\delta CO_{2}\to CeO_{2}+\delta CO$$

where *δ* denotes the nonstoichiometry—a measure of the oxygen exchange capacity and therefore of the fuel yield per cycle. For typical operating conditions of the reduction step at 1,500°C and 0.1 mbar, and the oxidation step at 900°C and 1 bar, thermodynamics predict *δ* = 0.04. Solar reactor concepts previously investigated for effecting the ceria redox cycle have included moving^8^, ^9^, ^10^, ^11^, ^12^ and stationary^13^, ^14^, ^15^ bulk structures, packed beds,^16^^,^^17^ moving beds,^18^^,^^19^ and aerosol flow^20^^,^^21^ of particles. Of special interest is the solar reactor concept based on a cavity-receiver containing reticulated porous ceramic (RPC) structures made of ceria,^22^^,^^23^ which provide efficient heat and mass transfer. Using an early prototype, the conversion of H_2_O and CO_2_ to renewable kerosene was demonstrated at the laboratory scale using a high-flux solar simulator.^24^ Recently, two identical solar reactors were operated at the focus of a solar parabolic concentrator for performing both redox steps of the thermochemical cycle simultaneously by alternating the concentrated solar input between them.^25^ While one solar reactor was performing the endothermic reduction step on sun, the second solar reactor was performing the exothermic oxidation step off sun, yielding a semi-continuous flow of syngas suitable for either methanol or FT synthesis. Stable outdoor operation was demonstrated for this solar fuel system, for which the mean solar radiative power input (*P*_solar_) through the solar reactor’s aperture was 5 kW.^25^

Despite recent advances, the scalability of the solar reactor remains a critical challenge to the commercialization of solar fuel production. The solar parabolic dish configuration is limited in size because of mechanical constraints due to wind and weight loads. Although multiple solar parabolic dishes may be deployed for scaling-up, a solar tower configuration features significant economy-of-scale advantages, as already seen for concentrated solar thermal power (CSP) plants,^26^ and will likely be seen for solar fuel plants as well. Ultimately, the solar reactor technology will have to be scaled up for a solar tower configuration. Here, we describe the design, fabrication, and testing of a 50-kW solar reactor and experimentally demonstrate, for the first time, the entire sun-to-fuel process chain from H_2_O and CO_2_ to kerosene in a solar tower configuration. This pioneer demonstration was realized within the framework of the EU Horizon 2020 project SUN-to-LIQUID.^27^ We evaluate and report the performance of the solar reactor—the cornerstone technology—based on five primary metrics, namely, reaction selectivity, syngas quality, fuel purity, energy efficiency, and material stability. The operation of a fully integrated solar tower fuel plant under intermittent solar radiation provides compelling evidence of the technical feasibility of the solar thermochemical technology for industrial scale implementation.

## Results and discussion

**Figure 1:**
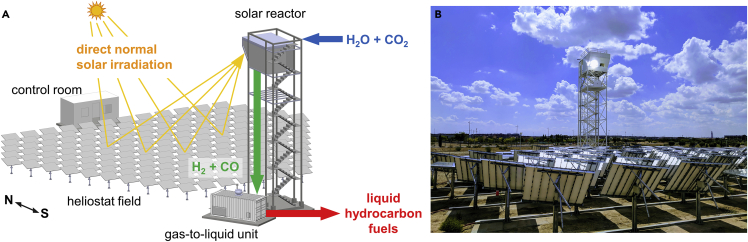
Overview of the solar tower fuel plant installed at IMDEA Energy (Spain) (A) Schematic of the solar tower fuel plant, encompassing the solar tower concentrating facility, the solar reactor, and the GtL unit. A heliostat field concentrates the direct normal solar irradiation onto a solar reactor mounted on top of the solar tower. The solar reactor co-splits H_2_O and CO_2_ and produces a specific mixture of H_2_ and CO (syngas), which in turn is processed to liquid hydrocarbon fuels using the FT-based GtL unit located next to the solar tower base. All sub-systems are operated from the control room. (B) Photograph of the solar tower fuel plant during operation.

The solar tower fuel plant, realized at IMDEA Energy in Spain, is depicted in Figure 1. It integrates three sub-systems: (1) the solar tower concentrating facility, (2) the solar reactor, and (3) the gas-to-liquid (GtL) unit. The solar concentrating facility consists of a solar tower with a south-facing heliostat field: an array of 169 sun-tracking spherical reflectors, each with an area of 3 m^2^, delivering a *P*_solar_ of about 50 kW into the 16-cm diameter aperture of the solar reactor, which corresponds to an average solar concentration ratio of approximately 2,500 suns, with a peak above 4,000 suns (1 sun is equivalent to a solar radiative flux of 1 kW/m^2^).^28^ The solar reactor is mounted on top of the solar tower at an optical height of 15 m, tilted 40° downward relative to the horizontal plane, and aimed at the power-weighted center of the heliostat field. On the ground next to the solar tower, the GtL unit is fully assembled inside a modular container. The experimental setup, peripheral components, and measurement instrumentation are described in detail in the supplemental information. The heliostat field is shown in the photograph of Figure S1. The solar reactor is described in experimental procedures.

**Figure 2:**
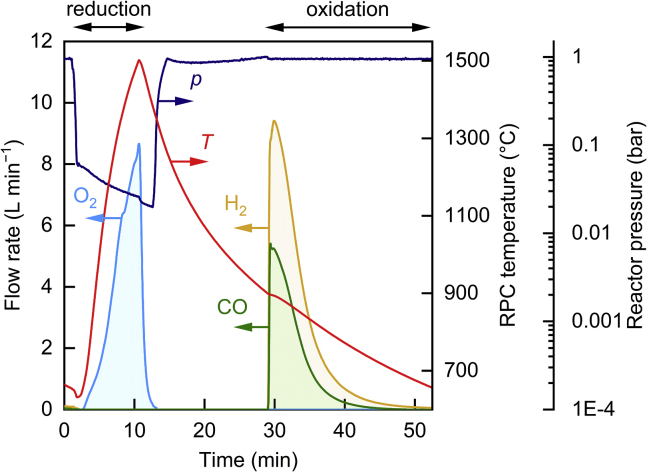
Temporal variations of the nominal RPC temperature, reactor pressure, and gaseous product (O_2_, CO, and H_2_) evolution rates during an exemplary redox cycle Experimental conditions during reduction: mean *P*_solar_ = 42.0 ± 6.2 kW; volumetric flow rate of Argon (V̇_Ar_) = 5.0 L min^−1^ at pressure (*p*) ≤ 70 mbar. Experimental conditions during oxidation: ṅ_H2O_ = 0.033 mol s^−1^, ṅ_CO2_ = 0.0074 mol s^−1^, at *p* ≈ 1 bar. Ceria RPC mass (*m*_RPC_) = 18.1 kg.

An exemplary redox cycle operated in a temperature/pressure-swing mode is shown in Figure 2, where the nominal RPC temperature, the reactor pressure, and the gas product flow rates of O_2_, CO, and H_2_ are plotted as a function of time. The experimental conditions and results of this run are summarized in Table 1. During the reduction step at an average *P*_solar_ = 42.0 ± 6.2 kW, and under vacuum conditions, the nominal RPC temperature rapidly increased up to the reduction end temperature (*T*_reduction,end_) of 1,502°C at a mean heating rate of about 100°C min^−1^. Accordingly, the rate of O_2_ evolution increased to a maximum of 8.7 ± 0.2 L min^−1^. Integrated over the entire reduction step, a total amount of 36.2 ± 0.7 L O_2_ was released, which, assuming all ceria reacted uniformly, corresponds to a specific oxygen exchange capacity of 0.002 L/g ceria and an average oxygen nonstoichiometry at the end of the reduction step of *δ* = 0.031. This indicates that the system approached thermodynamic equilibrium, consistent with pervious tests with the laboratory-scale reactor.^22^ At the end of the reduction step after 8.8 min, the solar input was interrupted (*P*_solar_ = 0), and the oxygen release rate rapidly decreased to zero, whereas the RPC naturally cooled down to the nominal oxidation start temperature (*T*_oxidation,start_) of 900°C within 18.3 min. Oxidation was initiated by simultaneously injecting H_2_O and CO_2_ at molar flow rates of ṅ_H2O_ = 0.033 mol s^−1^ and ṅ_CO2_ = 0.0074 mol s^−1^. Both H_2_ and CO production rates peaked shortly after at 9.4 ± 0.8 L min^−1^ and 5.4 ± 0.4 L min^−1^, respectively, and decreased monotonically until the ceria was fully re-oxidized after 24.0 min when the oxidation end temperature (*T*_oxidation,end_) reached 654°C. Integrated over the entire oxidation period, a total amount of 48.9 ± 3.9 L H_2_ and 24.4 ± 2.0 L CO was produced. Mass balance of both redox steps yields a corresponding molar ratio (H_2_ + CO):O_2_ = 2.03 ± 0.21, indicating full selectivity for the conversion of H_2_O to H_2_ and CO_2_ to CO. No side reactions or by-products were detected. Note that the molar ratio of the fed reactants reached H_2_O:CO_2_ = 4.5 because excess water was required to obtain the desired syngas quality for FT synthesis. For the exemplary run of Figure 2, H_2_:CO = 2.01 ± 0.35, which is suitable for FT synthesis.

**Table 1:** Experimental conditions and results of the exemplary solar redox cycle of Figure 2

Variable	Symbol	Value	Unit
Ceria RPC mass	*m*_RPC_	18.1	kg
Average solar power input during reduction	*P*_solar_	42.0 ± 6.2	kW
Solar power input during oxidation	N/A	0	kW
Reduction start temperature	*T*_reduction,start_	632	°C
Reduction end temperature	*T*_reduction,end_	1,502	°C
Oxidation start temperature	*T*_oxidation,start_	900	°C
Oxidation end temperature	*T*_oxidation,end_	654	°C
Ar flow rate during reduction	V̇_Ar_	5.0	L min^−1^
H_2_O flow rate during oxidation	*ṅ*_H2O_	0.033	mol s^−1^
CO_2_ flow rate during oxidation	*ṅ*_CO2_	0.0074	mol s^−1^
Reactor pressure during reduction	N/A	26–70	mbar
Reactor pressure during oxidation	N/A	atmospheric	N/A
Reduction duration	N/A	8.8	min
Duration of cooling-down	N/A	18.3	min
Oxidation duration	N/A	24.0	min
Cycle duration	N/A	51.1	min
Mean heating rate	N/A	98.9	°C min^−1^
Peak O_2_ evolution rate	N/A	8.7 ± 0.2	L min^−1^
Total amount of O_2_ released	N/A	36.2 ± 0.7	L
Average nonstoichiometry of ceria after reduction	δ	0.031 ± 0.001	N/A
Peak H_2_O evolution rate	N/A	9.4 ± 0.8	L min^−1^
Total amount of H_2_O produced	N/A	48.9 ± 3.9	L
Peak CO evolution rate	N/A	5.4 ± 0.4	L min^−1^
Total amount of CO produced	N/A	24.4 ± 2.0	L
Molar ratio (H_2_ + CO)/O_2_	N/A	2.03 ± 0.21	N/A
Molar ratio H_2_/CO	N/A	2.01 ± 0.35	N/A
Solar-to-syngas energy efficiency	*η*_solar-to-syngas_	4.1 ± 0.8	%

Besides reaction selectivity and syngas quality, an important performance indicator that particularly affects the economic viability of the process is the solar-to-syngas energy conversion efficiency (*η*_solar-to-syngas_), defined as the ratio of the calorific value of the syngas produced over the cycle to the sum of solar radiative energy input (*Q*_solar_, obtained by integrating *P*_solar_ over the cycle, $Q_{\text{solar}}=\int P_{\text{solar}}dt$) and additional parasitic energy inputs associated with inert gas consumption and vacuum pumping (see supplemental information for efficiency formulation; Figures S2 and S3 for details on the solar radiative power determination). The energy conversion efficiency depends primarily on the amount of syngas produced (H_2_ and/or CO) during the oxidation step, compared with the amount of solar energy required to release O_2_ during the reduction step. For the exemplary run of Figure 2, *η*_solar-to-syngas_ = 4.1 ± 0.8% at an average *P*_solar_ = 42.0 ± 6.2 kW. For pure CO_2_-splitting, *η*_solar-to-syngas_ = 5.6 ± 1.0% at an average *P*_solar_ = 55.8 ± 8.2 kW. From an operational perspective, the primary difference between these two reported efficiencies was *P*_solar_. A higher *P*_solar_ for the pure CO_2_-splitting run resulted in rapid heating and a shorter reduction cycle, which in turn led to lower *Q*_solar_ ($Q_{\text{solar}}=\int P_{\text{solar}}dt$= 20.1 MJ, versus 22.2 MJ for the co-splitting of H_2_O and CO_2_) and consequently higher *η*_solar-to-syngas_. On the other hand, the co-splitting run used excess water, which consumed part of *Q*_solar_ upon heating to *T*_reduction,end_ and led to lower *η*_solar-to-syngas_. Splitting pure H_2_O and pure CO_2_ in separate cycles and mixing the product gases H_2_ and CO can also be applied to obtain the syngas composition required for FT synthesis, eliminating the need for excess water during a co-splitting run.

These measured values of energy conversion efficiency were obtained without any implementation of heat recovery. Specifically, the sensible heat rejected during the temperature-swing redox cycling accounted for more than 50% of *Q*_solar_. This fraction can be partially recovered via thermocline heat storage, as demonstrated with a packed bed of Al_2_O_3_ spheres, which was able to recover half of the sensible energy stored for a temperature swing between 1,400°C and 900°C.^29^ Thermodynamic analyses indicate that sensible heat recovery could potentially boost *η*_solar-to-syngas_ to values exceeding 20%.^3^^,^^4^ Furthermore, it was evident from the temperature distribution across the RPC that the reaction extent was not uniform. Heat-transfer modeling estimated a temperature difference between the directly irradiated front and the back surface of the ceria RPC to exceed 200°C.^30^ This is mainly caused by the exponential decay of transmitted radiation (Bouguer’s law) observed for a RPC of uniform porosity, resulting in a significant temperature gradient across the RPC thickness. The ratio between the actual released O_2_ and the amount of O_2_ that could theoretically be released if all ceria mass would have reached uniform temperature at the end of the reduction step is estimated to be approximately 0.5. This ratio can be increased by modifying the radiation attenuation, for example, by manufacturing hierarchically ordered porous structures with a step-wise porosity gradient, which can augment the volumetric radiative absorption and lead to a more uniform temperature distribution and, ultimately, higher efficiencies.^31^^,^^32^

Note that *η*_solar-to-syngas_ only considers the performance of the solar reactor sub-system. The energy efficiency of the entire solar fuel plant should also consider the performance of the other two sub-systems upstream and downstream of the solar reactor, namely, the optical efficiency of the solar concentrating tower facility (hoptical)) and the energy efficiency of the GtL unit (*η*_GtL_). *η*_optical_ depends on the heliostat layout, geometry, reflectivity, tracking accuracy, shading/blocking, attenuation, and cosine losses and can reach values up to 70% while keeping a mean solar flux concentration of 2,500 suns over the solar reactor’s aperture, provided radiation spillage is collected and used (for example to preheat gaseous reactants).^28^ *η*_GtL_ depends mainly on the targeted product, catalyst, and syngas composition. When targeting methanol synthesis and assuming autothermal operation, 90% mass conversion, and accounting for the equivalent thermal energy penalty for syngas compression to 60 bars, *η*_GtL_ was estimated to be 75%.^25^ When targeting FT synthesis, *η*_GtL_ further depends on the definition of mass conversion, since several valuable products (e.g., kerosene or diesel) can be co-generated.

**Figure 3:**
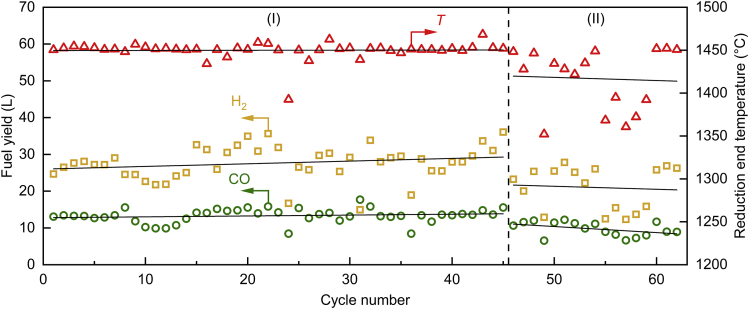
Multiple consecutive redox cycles Nominal ceria RPC temperature at the end of the reduction step and total amounts of produced H_2_ and CO per cycle for 62 consecutive redox cycles, yielding 5,191 ± 364 L of syngas with a composition 31.8% ± 3.2% H_2_, 15.2% ± 2.4% CO, and 53.0% ± 3.6% CO_2_ (H_2_O condensed). Linear fits are shown. L denotes standard liters.

Stable performance of the solar reactor over a large number of redox cycles is essential for any potential commercial application. The morphological stability of a similar ceria RPC was previously demonstrated with 227 consecutive redox cycles in a 4-kW solar reactor^24^ and with 500 consecutive cycles in an infrared.^22^ For the 50-kW solar reactor in this study, 62 consecutive redox cycles were performed during a dedicated and continuous fuel production campaign. A representative cycle is shown in Figure S4. The cycles were conducted over a period of 9 days, 6–8 cycles/day (except for one day when cycle #24 was interrupted by clouds), with an average duration of 53 min/cycle and a total experimental time of 55 h (see also the operational strategy described in Figure S5 during a representative day run, including a heating phase, a pre-cycle, consecutive cycling, and a natural cooling phase). Figure 3 shows the nominal RPC temperature at the end of the reduction step and the total amounts of H_2_ and CO produced per cycle for all 62 cycles. During the first 45 cycles (region I), the targeted *T*_reduction,end_ of 1,450°C ± 18°C was reached for all cycles (except for cycle #24), yielding a relatively constant fuel production. However, during the last 17 cycles (region II), *T*_reduction,end_ varied as several cycles were stopped early due to critical high temperatures (>1,500°C) measured at the back of the RPC cavity. These temperature variations from cycle to cycle directly resulted in variations of the oxygen released and, consequently, the fuel amounts produced. Although an effort was made to maintain constant operating conditions for all consecutive cycles, temporal variations of the direct normal irradiance (DNI) and of the tracking of the heliostat field resulted in varying *P*_solar_ and, consequently, in temperature and product gas fluctuations. In more than 90% of the cycles, the trend in CO and H_2_ yield was as expected, i.e., increasing together or decreasing together with higher or lower reduction temperatures, respectively. For the few cycles where the expected trend is not observed, the deviation is minimal, presumably caused by temporal and/or spatial variations of the RPC temperature affecting the reduction extent of ceria (δ) and in turn its oxidation with H_2_O and CO_2_. Degradation of the ceria RPC caused by the local formation of cracks was observed (see supplemental information, in particular Figures S6 and S7), presumably caused by the critical temperatures measured at the back of the RPC cavity. Nonetheless, the interlocking design of the RPC bricks ensured the integrity of the cavity assembly. Overall, 5,191 ± 364 L of syngas were produced with a composition of 31.8% ± 3.2% H_2_, 15.2% ± 2.4% CO, and 53.0% ± 3.6% unreacted CO_2_, whereas the unreacted H_2_O was condensed. The corresponding molar ratio of H_2_:CO was 2.1. Around 91% of the produced syngas was subsequently processed on-site by the GtL unit, yielding a liquid phase containing 16% kerosene and 40% diesel, and a wax phase containing 7% kerosene and 40% diesel. See Figure S8 for additional details on the FT product distribution.

In summary, the technical feasibility of the entire thermochemical process chain to produce solar liquid hydrocarbon fuels from H_2_O and CO_2_ has been demonstrated with a pilot-scale solar tower fuel plant that integrates, in series, the three main sub-systems, namely: the solar concentrating tower, the solar reactor, and the GtL unit. The solar reactor produced syngas with selectivity, purity, and quality suitable for FT synthesis. Although the *η*_solar-to-syngas_ is still in the single digits, it has the potential to reach competitive values of over 20% by recovering rejected heat during the temperature-swing redox cycle and by improving the volumetric absorption of the porous structures. The ceria RPC remains the most critical component of the solar reactor and further progress with the manufacturing of mechanically robust porous structures remains essential. Alternative material compositions, e.g., perovskites^33^ or aluminates,^34^ may yield sufficient redox capacity at lower, more moderate temperatures or under isothermal conditions. Adjustments to the cavity geometry and concentrating optical system, i.e., by incorporating a secondary compound parabolic concentrator (CPC), can further improve the uniformity of the radiative flux distribution within the cavity and consequently alleviate the thermal stressing. One approach to scaling up the solar fuel plant would be to use an array of solar cavity-receiver modules arranged side-by-side, each attached to hexagon-shaped CPC in a honeycomb configuration. The solar tower fuel plant described here represents a viable pathway to global-scale implementation of solar fuel production. If CO_2_ is further captured from the air or derived from a biogenic source, the resulting drop-in hydrocarbon fuels, e.g., kerosene, can be considered carbon neutral.^25^^,^^35^ Life-cycle assessment and economic feasibility of the complete fuel process chain, analogous to the pathway demonstrated in this study, as well as benchmarking vis-à-vis alternative approaches to the production of drop-in fuels using solar energy, were discussed in previous publications.^25^^,^^36^^,^^37^

## Experimental procedures

### Resource availability

#### Lead contact

Further information and requests for resources should be directed to Aldo Steinfeld, aldo.steinfeld@ethz.ch.

#### Materials availability

This study did not generate new unique materials.

## Data Availability

**Figure 4:**
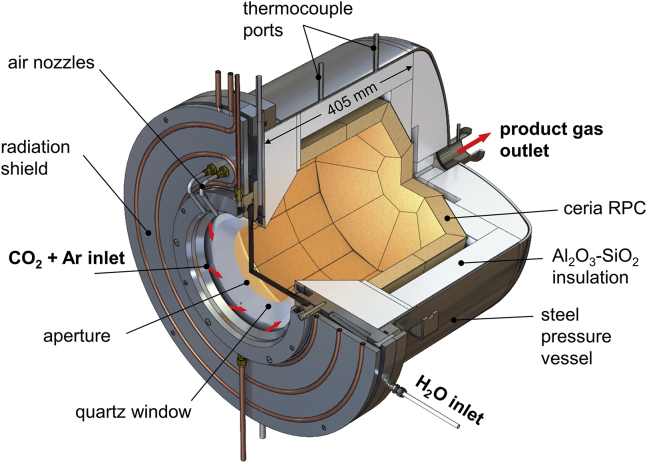
Schematic of the solar reactor for splitting H_2_O and CO_2_ via the ceria-based thermochemical redox cycle It consists of a cavity-receiver containing a ceria RPC structure directly exposed to concentrated solar radiation entering through a windowed circular aperture. During the reduction step, the RPC is exposed to the high solar fluxes; O_2_ evolves. During the oxidation step, reacting gases CO_2_ and H_2_O enter via tangential inlet ports at the front and flow across the porous RPC; syngas is formed. Product gases (O_2_ during the reduction step, syngas during the oxidation step) exit via an axial port at the rear of the vessel. Schematic of the solar reactor for splitting H_2_O and CO_2_ via the ceria-based thermochemical redox cycle It consists of a cavity-receiver containing a ceria RPC structure directly exposed to concentrated solar radiation entering through a windowed circular aperture. During the reduction step, the RPC is exposed to the high solar fluxes; O_2_ evolves. During the oxidation step, reacting gases CO_2_ and H_2_O enter via tangential inlet ports at the front and flow across the porous RPC; syngas is formed. Product gases (O_2_ during the reduction step, syngas during the oxidation step) exit via an axial port at the rear of the vessel. The solar reactor geometry was determined by applying CFD simulations.^30^^,^^38^ Key scaling parameters and considerations when moving from the 4-kW lab-scale prototype^22^ to the 50-kW reactor design included: (1) determining the aperture size paired to a given heliostat field in order to achieve a mean solar flux over the aperture of 2,500 suns; (2) selecting a cavity geometry that gives an apparent absorptivity approaching 1; (3) determining the RPC exposed surface area to maintain an incident flux of 125 suns; (4) arranging the inlet/outlet gas ports to achieve uniform and stable fluid flow across the RPC; (5) increasing the RPC thickness and number of facets to support a larger interlocking brick structure; and (6) maintaining the RPC porosity without sacrificing structural integrity. The dual-scale interconnected porosity (mm and μm size pores) provided volumetric radiative absorption during the reduction step and faster reaction kinetics during the oxidation step.^39^ Engineering details are provided in the supplemental information. Solar-produced syngas exits the solar reactor sub-system at the top of the tower, and after condensing unreacted H_2_O and passing through in-line gas analysis, flows at near ambient pressure down the tower, where it is pressurized and stored in a 50-L buffer tank at 30–150 bar. The GtL unit controller automatically draws syngas from the buffer tank to perform the FT catalytic conversion in its cobalt-based packed-bed reactor at 30 bar and 210°C. The FT synthesis requires a syngas with H_2_:CO molar ratio of around 2.15,^40^ which the solar reactor sub-system is able to match very closely by varying the mass flow rate of reactants H_2_O and CO_2_ during the oxidation step. The resulting long-chain hydrocarbons are collected in a downstream vessel for sampling and analysis. Despite the intermittent nature of the solar resource, the buffer tank enables the GtL unit to be operated with any desired production schedule, ranging from 24/7 slow and steady operation to short duration and high production rate operation.

The main data supporting the findings of this study are available within the paper and its supporting documentation. Source data are available with this paper. The solar reactor was based on a previous laboratory-scale design,^22^ which was scaled up from 4 kW to a nominal 50 kW of *P*_solar_, which corresponds to a scaling factor of 12.5. Its configuration is schematically shown in Figure 4. It consists of a well-insulated cavity-receiver with a 16-cm diameter circular aperture where concentrated solar radiation enters. The aperture is sealed with a transparent quartz window mounted on a refrigerated radiation shield and actively cooled from the outside by air nozzles. The cavity contains a cylindrical structure of interlocking RPC bricks made of ceria (see Figure S9). With this arrangement, the RPC bricks are directly exposed to concentrated solar radiation coming from the heliostat field, providing efficient radiative heat transfer directly to the reaction site. During the oxidation step, reacting gases CO_2_ (purity 99.9%) and H_2_O (deionized) enter the reactor via tangential inlet ports at the front and flow across the porous RPC; product gases (O_2_ during the reduction step, syngas during the oxidation step) exit via an axial port at the rear of the vessel. A lower purity of CO_2_ feedstock, i.e., containing 1%–2% air as might be obtained by direct air capture, would not significantly influence the performance of the solar reactor because N_2_ is inert and O_2_ would be consumed by oxidizing the reduced ceria RPC.^25^ A detailed process flow schematic is shown in Figure S10.

## Supplemental information

Document S1. Supplemental experimental procedures, Figures S1–S10, and supplemental references

Data S1. Data for nominal RPC temperature, reactor pressure, and gaseous products (O_2_, CO, and H_2_) evolution rates versus time during an exemplary redox cycle

Data S2. Data for nominal RPC temperature at the end of the reduction step and total amounts of produced H_2_ and CO per cycle for 62 consecutive redox cycles

Data S3. Data for solar radiative power input measured with the water-calorimeter and the FMAS and the nominal RPC temperature versus time for an exemplary redox cycle

Data S4. Data for nominal RPC temperature and product gas concentrations (O_2_, H_2_, CO, and CO_2_) versus time for an exemplary redox cycle

Data S5. Data for solar radiative power input and nominal RPC temperature versus local time for a representative experimental run

Document S2. Article plus supplemental information
