# Supplementary material for: A solar tower fuel plant for the thermochemical production of kerosene from H2O and CO2
Source: Joule. 2022 Jul 20;6(7):1606–16. doi: 10.1016/j.joule.2022.06.012 (PMC9332358; doi:10.1016/j.joule.2022.06.012)
Supplement: Document S1. Supplemental experimental procedures, Figures S1–S10, and supplemental references [file mmc1.pdf]

Joule, Volume *6*

Supplemental information

A solar tower fuel plant

for the thermochemical production

# of kerosene from H_2O and CO_2

Stefan Zoller, Erik Koepf, Dustin Nizamian, Marco Stephan, Adriano Patané, Philipp Haueter, Manuel Romero, José González-Aguilar, Dick Lieftink, Ellart de Wit, Stefan Brendelberger, Andreas Sizmann, and Aldo Steinfeld

Supplemental Experimental Procedures

Solar Tower Concentrating Facility

The heliostat field is shown in the photograph of Figure S1 (left). It consists of 169 heliostats, 3 m^2 each, arranged in a 14-row cornfield layout with focal lengths of 20 m (rows 1-8) and 30 m (rows 9-14).[41] The row and azimuthal spacing between the single facet heliostats are 2.25 and 2.60 m respectively, resulting in 47% land use, and the distance from the tower to the first row of heliostats is only 4 m. Each heliostat (Figure S1, right) is mounted on a 1.4 m-height pedestal pole and tracks the sun according to a tilt-roll or fixed horizontal tracking with the help of two rectilinear actuators having a roll angle range ± 100°, elevation angle range 20-90º (90º corresponding to the horizontal stow position facing to zenith), with tracking accuracy < 0.1º. Cold mechanical bending was applied to 3 mm-thick rectangular mirrors (dimensions 1605x1900x3 mm, total reflectivity 94.3%), bonded to a metallic frame and pre-conformed by gravity sagging onto a master model to obtain a spherical curvature. The mean beam quality, excluding sunshape, is 2.5 mrad. Based on ray-tracing analysis for spring equinox, summer, and winter solstice, this facility delivers a total solar radiative power of about 250 kW, of which at least 50 kW are incident within a 16-cm diameter circular target on top of the solar tower throughout the entire year. The optical efficiency η_{optical} can reach values exceeding 70% provided radiation spillage is collected and used.[41]

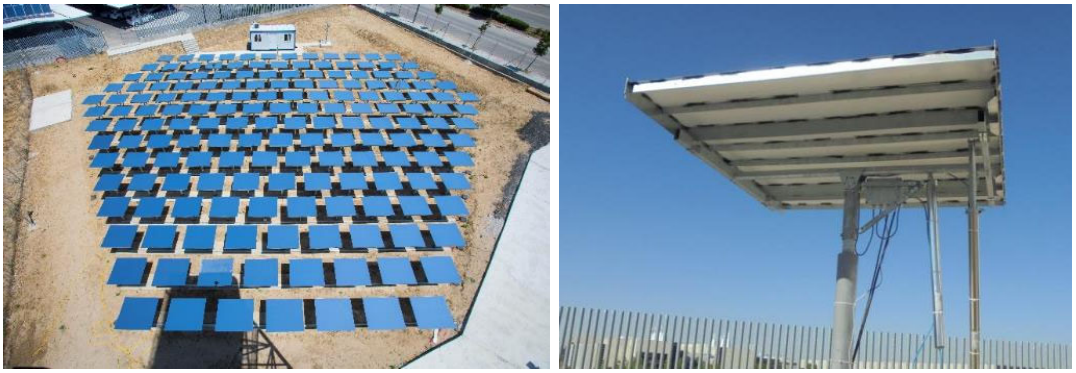

Figure S1. Photographs of the heliostat field. *Left*: Aerial view of the heliostat layout from the top of the solar tower. *Right*: Lateral view of a 3 m^2 heliostat with two rectilinear actuators.

The Solar-to-Syngas Energy Efficiency

The solar-to-syngas energy conversion efficiency $\eta_{\text{solar-to-syngas}}$ is defined as the ratio of the calorific value of the syngas produced over the cycle to the sum of solar radiative power input Q_{solar} (obtained by integrating P_{solar} over the cycle) and any additional parasitic energy inputs (in our case: energy inputs associated with vacuum pumping and inert gas consumption):[42,43]

$$\eta_{\text{solar-to-syngas}} = \frac{Q_{\text{syngas}}}{Q_{\text{input}}} = \frac{Q_{\text{syngas}}}{Q_{\text{solar}} + Q_{\text{pump}} + Q_{\text{inert}}} \tag{1}$$

The energy content of the fuel produced, Q_{syngas}, is given by:

$$Q_{\text{syngas}} = \Delta H_{\text{H}_2} \cdot \int r_{\text{H}_2} dt + \Delta H_{\text{CO}} \cdot \int r_{\text{CO}} dt \tag{2}$$

where ΔH_{H_2} and ΔH_{CO} are the higher heating values of H_2 and CO ($\Delta H_{\text{H}_2} = 286$ kJ mol^{-1} and $\Delta H_{\text{CO}} = 283$ kJ mol^{-1}) and $\int r_{\text{H}_2} dt$ and $\int r_{\text{CO}} dt$ are the measured molar rates of produced H_2 and CO integrated over the duration of the reduction step. Q_{solar} is the total solar energy input to the solar reactor integrated over the reduction step, as concentrated solar energy is only delivered during the endothermic reduction, and is defined as:

$$Q_{\text{solar}} = \int P_{\text{solar}} dt \tag{3}$$

where P_{solar} is the solar radiative power input through the reactor's aperture, taking into account absorption and reflection losses at the quartz window, and was measured using the methodology described above. Q_{pump} and Q_{inert} are the energy penalties associated with vacuum pumping and the consumption of the inert gas (Ar) during the reduction step, respectively. The vacuum pumping energy is calculated as the thermodynamic minimum pumping work divided by a heat-to-work energy efficiency, $\eta_{\text{heat-to-work}}$ (assumed to be 0.4),[44,45] and a pressure dependent vacuum pumping efficiency, η_{pump}, according to

$$Q_{\text{pump}} = \frac{1}{\eta_{\text{heat-to-work}}} \cdot \int \frac{R \cdot T_{\text{pump}}}{\eta_{\text{pump}}(p(t))} \cdot \dot{n}(t) \cdot \ln\left(\frac{p_{\text{atm}}}{p(t)}\right) dt \tag{4}$$

where $\dot{n}(t)$ is the molar gas flow rate pumped out of the reactor, consisting of the inert gas (Ar) injected to the reactor, the O_2 released by the ceria, and the gas evacuated from the reactor during transient change of pressure, R is the universal gas constant (R = 8.314 J K^{-1} mol^{-1}), T_{pump} is the

pump temperature (assumed 298.15 K), and p_{atm} and p are atmospheric and reactor pressure, respectively. The pumping efficiency is based on the analysis of a multi-stage industrial vacuum pump arrangement from Pfeiffer vacuum:[45]

$$\eta_{\text{pump}}\left(p_{\text{reactor}}(t)\right) = 0.07 \cdot \log\left(\frac{p(t)}{p_{\text{atm}}}\right) + 0.4 \tag{5}$$

The energy required for the separation of the inert gas is defined as:

$$Q_{\text{inert}} = \frac{1}{\eta_{\text{heat-to-work}}} E_{\text{inert}} \int r_{\text{inert}} dt \tag{6}$$

where E_{inert} is the work required for inert gas separation (assumed 20 kJ per mole)[46] and r_{inert} is the flow rate of the inert gas during reduction. Note that $\eta_{solar\text{-}to\text{-}syngas}$ is weakly dependent on the assumptions used for the calculation of the two energy penalties because Q_{solar} is roughly two orders of magnitude larger than Q_{pump} and Q_{inert}. The accuracy of the measured P_{solar} and produced fuel volumes were considered for estimating the uncertainty in the value of $\eta_{solar\text{-}to\text{-}syngas}$, which was dominated by the uncertainty in the energy input (±15%) mainly resulting from the position offset between the aperture of the calorimeter for power measurement and the aperture of the solar reactor.

Solar Radiative Flux Measurement

Accurately measuring the solar radiative power entering the solar reactor through its aperture, P_{solar}, is crucial for the determination of the solar-to-syngas energy conversion efficiency, $\eta_{solar\text{-}to\text{-}syngas}$. P_{solar} depends mainly on the direct solar normal irradiance (DNI) incident on the heliostat field, the day of the year, the time of the day, and the optical efficiency of the solar concentrating facility $\eta_{optical}$. The measurement of P_{solar} employed two different methods and devices: (1) A water-calorimeter and (2) a flux measurement acquisition system (FMAS). The arrangement of the two devices in the solar tower is depicted in Figure S2 (left). The water-calorimeter was positioned next to the solar reactor at the same optical height and with the same inclination angle (but shifted 1.6 m to the east) of the solar reactor. Outside the fronts of the solar reactor and water-calorimeter, a porous Al_2O_3 insulation board was installed as a passive thermal shield to block spilled radiation. The optical measurement by the FMAS was based on recording the intensity flux map of diffusely

reflected radiation.[47] The FMAS setup consisted of a water-cooled, Al_2O_3 plasma-coated Lambertian target mounted on a linear belt for alignment in front of either the solar reactor or the water-calorimeter, and a CCD camera with a telephoto lens mounted in the control room.[48] The CCD camera was calibrated using a Gardon-type flux gauge embedded in the target. The power was calculated by integrating the compiled flux map over a 16 cm-dia. circular area. A representative solar flux map generated with the FMAS is shown in Figure S2 (right). Integration of the solar flux over a 16 cm-dia. circular target gives P_{solar} = 50.4 kW, with a peak flux of 2932 kW m^{-2}. The FMAS accuracy is estimated to be ±6%, due primarily to the position offset relative to the aperture plane.[48] When not in use, the FMAS target was held in a standby position between the solar reactor and the water-calorimeter. The water-calorimeter was designed to closely approximate the geometric specifications of the aperture of the solar reactor. The outer cavity shell and the front section are identical to the respective parts of the solar reactor. The calorimeter's cavity contains approximately 20 meters of coiled copper tubing coated with a black (high absorptivity) enamel paint and is lined with rock wool thermal insulation. The water flow rate was measured with an electromagnetic flow meter (Endress+Hauser, Promag 33). Inlet and outlet water temperatures were recorded with four-wire Pt100 resistance thermometers. Using the measured temperature difference between inlet and outlet, combined with the mass flow rate of water, P_{solar} was directly calculated as the energy absorbed by the water flow. Heat losses by radiation, conduction and convection, estimated using an in-house Monte Carlo ray tracing and a simplified analytical model, accounted for less than 1% of P_{solar} at a solar radiative power input of 50 kW. Note that these calorimeter measurements included absorption and reflection losses at the quartz window. The accuracy was specified as ±2% and was driven primarily by the uncertainties in the temperature and water flow rate measurements. Due to the water-calorimeter's higher accuracy relative to the FMAS, the water-calorimeter measurements were applied to determine P_{solar}, which in turn was used to calculate the solar-to-syngas energy efficiency, $\eta_{solar\text{-}to\text{-}syngas}$. The FMAS measurements were used to estimate the solar flux distribution as well as P_{solar} at the aiming point of the solar reactor.

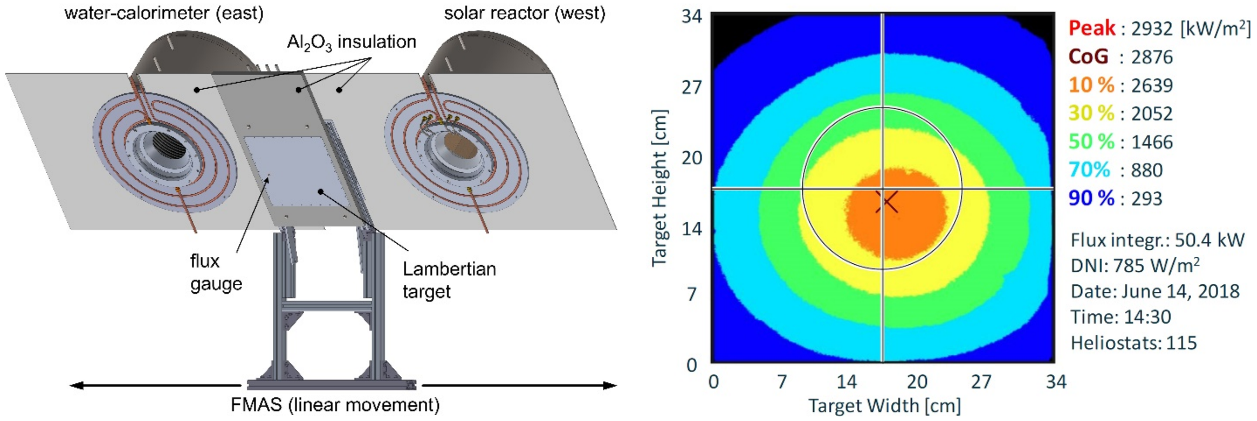

Figure S2. Details of the solar flux measurement system. *Left*: Scheme of the solar radiative power measurement installation in the solar tower. The Lambertian target of the flux measurement acquisition system (FMAS) moves on a linear system, which allows alignment in front of either the water-calorimeter or the solar reactor. *Right*: A representative solar flux map taken with the flux measurement acquisition system (FMAS). Reproduced from 'Koepf et al., 2019, Liquid fuels from concentrated sunlight: An overview on development and integration of a 50 kW solar thermochemical reactor and high concentration solar field for the SUN-to-LIQUID project', with the permission of AIP Publishing.[49]

The experimental procedure to determine P_{solar} is indicated in Figure S3 during a typical reduction step of the redox cycle. It shows the nominal RPC temperature in the solar reactor (red, right y-axis) and P_{solar} measured with the water-calorimeter (black, left y-axis) as well as with the FMAS (blue and orange data points, left y-axis) at two positions, in front of the water-calorimeter and in front of the solar reactor. The measurement procedure is as follows: first, the heliostat field is aimed at the water-calorimeter and the first FMAS measurement is taken in front of the water-calorimeter. The FMAS target is then moved to its standby position, and the concentrated solar radiation immediately enters the water-calorimeter, which approaches approximate thermal steady-state conditions within 2 minutes due to its low thermal mass and the high thermal conductivity of the copper tubing. After that, the heliostat field is aimed at the solar reactor and the second FMAS measurement is taken in front of the solar reactor. When the FMAS target is moved back to its standby position, the concentrated solar radiation is finally delivered to the solar reactor and the reduction cycle is started. When $T_{reduction,end}$ is reached, typically 1450°C, the measurement process is repeated, but in reverse. In the example of Figure S3, the steady-state P_{solar} measured by the water-calorimeter at the beginning and end of the reduction step were 39.9 and 41.3 kW, respectively. The slight increase is mainly due to a slight change in tracking errors, as changes in DNI were insignificant during the short reduction period (typically less than 15 min.). Therefore, a linear change of P_{solar} was assumed during reduction and the calculated mean value

during the reduction step was 40.6±6.0 kW. The measurement error is conservatively estimated by addition of the measurement inaccuracy of the water-calorimeter (2%) and the inaccuracy imposed by the position offset between the solar reactor aperture and water-calorimeter aperture (13%, calculated from the FMAS measurements). The grey area in Figure S3 corresponds to the total solar energy input to the solar reactor Q_{solar}, obtained by integrating P_{solar} over the duration of the reduction step. The relative difference between FMAS measurements at the two positions was used to evaluate the inaccuracy imposed by measuring P_{solar} at the position of the water-calorimeter (estimated at 13% of P_{solar}).

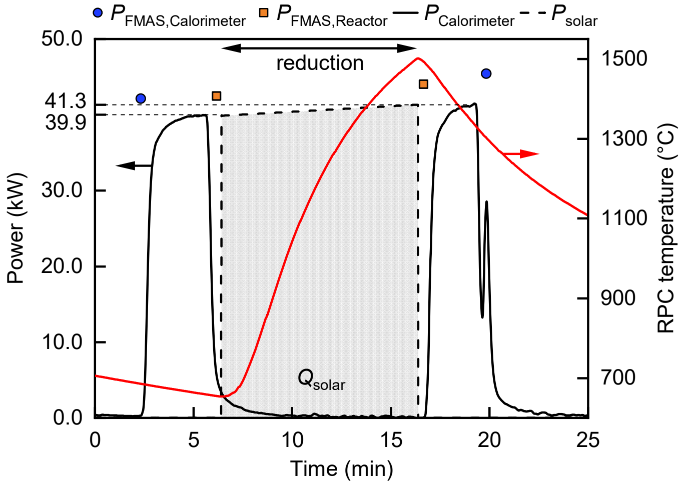

Figure S3. Temporal variation of the solar radiative power input P_{solar} measured by the water-calorimeter (black, left y-axis) and the nominal RPC temperature (red, right y-axis) during a representative reduction step of the redox cycle. Also indicated is the FMAS measurement at two positions: in front of the water-calorimeter and in front of the solar reactor (blue and orange data points, left y-axis). The grey area represents the total solar energy input Q_{solar}, i.e. the integral of P_{solar} over the duration of the reduction step.

Consecutive Redox Cycling

Figure S4 shows the nominal RPC temperature and the concentrations of O_2, H_2, CO and CO_2 measured in the product gas mixture exiting the solar reactor (after condensing unreacted H_2O) for a representative cycle during the campaign with 62 consecutive redox cycles. For all cycles, the reactor was evacuated to a vacuum pressure of less than 100 mbar before the solar radiative power was applied to heat the ceria RPC up to the target $T_{reduction,end}$ of 1450 °C. Shortly after the target temperature was reached, the reactor was slowly re-pressurized with a mixed flow of H_2O and CO_2. Once the ceria RPC temperature naturally cooled down to $T_{oxidation,start}$ of 900 °C, a mixture of 0.039 mol s^{-1} of H_2O and 0.0074 mol s^{-1} of CO_2, corresponding to a molar feeding ratio of 5.2,

was fed into the reactor. Oxidation was stopped when the measured CO_2 concentration approached 80%, to limit the amount of residual CO_2 in the collected syngas. For the exemplary cycle shown in Figure S4, this corresponds to an oxidation time of 6.8 min during which a total amount of 29.5±2.4 L H_2 and 13.3±1.6 L CO was produced.

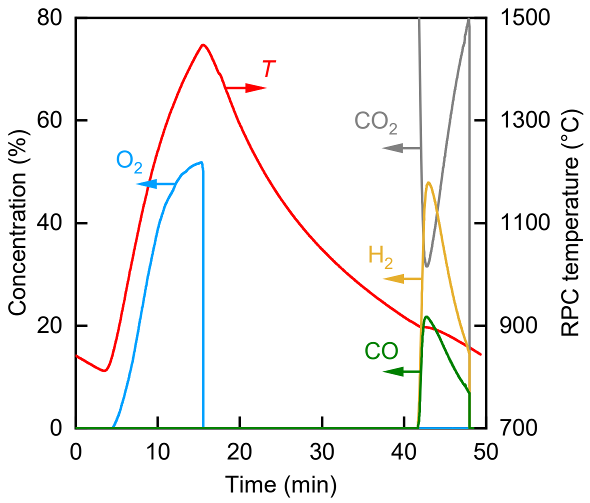

Figure S4. Nominal RPC temperature and concentrations of O_2, H_2, CO and CO_2 in the product gas exiting the solar reactor for a representative cycle during the long-term operation of the solar reactor. Experimental conditions during reduction: P_{solar} = 37.9±5.7 kW, $\dot{V}_{Ar}$ = 5.0 L min^{-1} at $p \leq 100$ mbar. Experimental conditions during oxidation: $\dot{n}_{H2O}$ = 0.039 mol s^{-1}, $\dot{n}_{CO_2}$ = 0.0074 mol s^{-1}, at $p \approx 1$ bar. Ceria RPC mass m_{RPC} = 21.3 kg.

Representative Experimental Run

The procedure during a typical experimental run consisted of a heating phase, a pre-cycle, consecutive cycling, and a natural cooling phase, as indicated in Figure S5. In this figure, the solar radiative power input to the reactor, P_{solar}, is shown in black (left y-axis) and the nominal RPC temperature is shown in red (right y-axis). The purpose of the heating and pre-cycle stages was to carefully bring the solar reactor up to operating temperature in the first cycle to avoid unnecessary thermal stresses. The first cycle was initiated by evacuating the solar reactor using the vacuum pumps and focusing a defined number of heliostats onto its aperture. To protect the quartz window from deposition of sublimated ceria and to govern the fluid flow when operating under vacuum conditions, an Ar flow rate of 5 L min^{-1} was introduced to the reactor directly behind the window. When $T_{reduction,end}$ was reached (typically 1450 °C), the reduction step was terminated by placing the target of the FMAS in front of the reactor (thereby effecting P_{solar} = 0), and letting the solar reactor cool down. The number of heliostats in operation did not change during a single reduction

step. We typically observed an increase of P_{solar} with time for cycles before solar noon (first 4 cycles of Fig. S7) and a decrease of P_{solar} with time for cycles after solar noon (last 2 cycles of Figure S5) due to smaller optical errors of the heliostat field close to solar noon. However, the difference in P_{solar} between what is measured at the start and at the end of a reduction step was small, typically less than 5% of P_{solar}, and did not affect the rate of heating. The reactor was then re-pressurized to atmospheric pressure with a mixture of H_2O and CO_2. Once the nominal RPC temperature decreased to $T_{oxidation,start}$ of 900 °C, H_2O and CO_2 were simultaneously introduced at constant rates, reacting with the reduced ceria and producing a mixed flow comprised of H_2, CO and unreacted CO_2 and H_2O, the latter removed via the condenser tubes. After the last redox cycle was terminated, the solar reactor naturally cooled down, typically approaching ambient temperature the morning of the next day. Up to eight consecutive cycles (including the pre-cycle) were performed per day with a typical cycle duration of around 50 minutes.

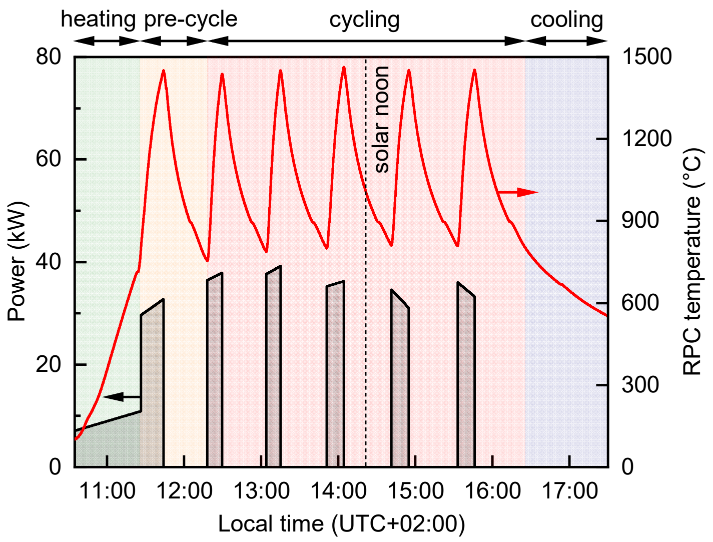

Figure S5: Operation strategy for the solar reactor during a representative experimental run, including a heating phase, a pre-cycle, consecutive cycling, and a natural cooling phase. The solar radiative power input, P_{solar}, is plotted in black on the left y-axis, and the nominal RPC temperature is plotted in red on the right y-axis.

Stability of Ceria RPC

The gradual structural degradation of the ceria RPC cavity can be observed in Figure S6. It shows the back of the ceria RPC cavity at three different stages: (a) before cycling, (b) after 30 completed redox cycles, and (c) after 46 redox cycles. Typically, after only a few cycles, we observe the formation of cracks on the ceria RPC bricks, some of them along their entire length. These cracks

presumably resulted from stresses induced by thermal and chemical expansion of ceria during the temperature swings. However, because of the self-supporting compression design, the cavity remained intact, notably without showing signs of degrading thermochemical performance. Over time, cracks can grow and even eventually lead to some degree of performance degradation. After 46 cycles, cracking at the back of the ceria RPC cavity and pieces falling to the floor of the cavity was observed, attributed to hot spots due to uneven distribution of the incoming solar radiation within the cavity walls. Note that the dark spot at the center left position in Figure S6 (c) is not caused by damage of the material but by incomplete re-oxidation of the ceria. Generally, it can be concluded that critical damage to the RPC can be avoided, possibly entirely, by ensuring a homogenous flux distribution on the RPC cavity walls.

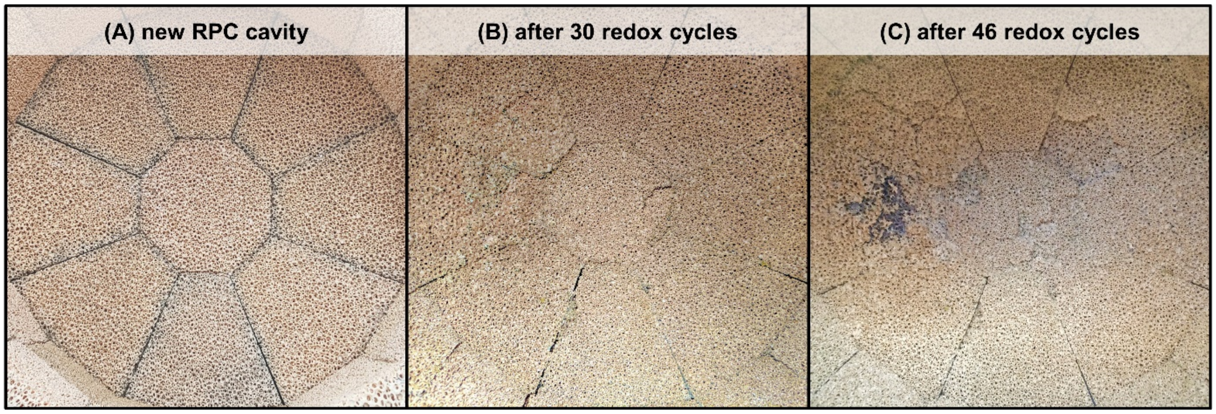

Figure S6. Photographs of the back of the ceria RPC cavity at three different stages of long-term usage: (A) the new RPC cavity after installation in the solar reactor; (B) after 30 consecutive redox cycles; and (C) after 46 redox cycles. Even with visible cracks, the interlocking cavity remains generally stable.

The effect of extended cycling operation on the morphological stability of the ceria RPC was assessed by comparing a newly produced RPC sample to samples extracted from two different positions within the RPC cavity after 62 consecutive redox cycles. Figure S7 shows scanning electron microscope (SEM) images of a RPC strut surface (top row) and cross section (bottom row) from (a) an unreacted RPC sample, (b) a sample extracted from the outer perimeter of the front lateral ring of RPC bricks (facing the reactor insulation) after 62 cycles, and (c) a sample extracted from the directly irradiated inner perimeter of the front lateral ring of RPC bricks after 62 cycles. For both samples extracted after cycling, larger grain sizes can be observed locally, while the grain size distribution is more uniform for the unreacted RPC. This could partly explain the degradation of the RPCs with increasing cycle number and increasing brittleness of the parts,

as the fracture strength of ceramic materials generally decreases with increasing grain size. The open µm-size porosity does not degrade with thermochemical cycling. This is consistent with previous studies confirming that the dual-scale porosity, both in the mm and µm scale, was preserved after 500 consecutive cycles, while cracks within the grains were observed.[42] Note that RPC manufacturing by the replication method is sensitive to various parameters such as the ceria particle size distribution, slurry composition, pore forming concentration, and sintering protocol, which can affect the structural integrity of the RPC. An additional difference can be observed between the surfaces of the directly irradiated inner perimeter and the outer perimeter because of the higher temperatures attained by the former, as a significant temperature gradient through the thickness of the RPC resulted from the exponential decay of the radiation intensity with penetration depth. For industrial scale operation of the solar reactor, one would need to consider the impact of, and ability to, remove, replace, and recycle the ceria RPC bricks. Given the modular nature of the scalable concept comprised of an array of solar receiver/reactors, such a replacement and recycling strategy could be implemented without major interruption, while minimizing or even eliminating the need for fresh ceria as a feedstock into the process.

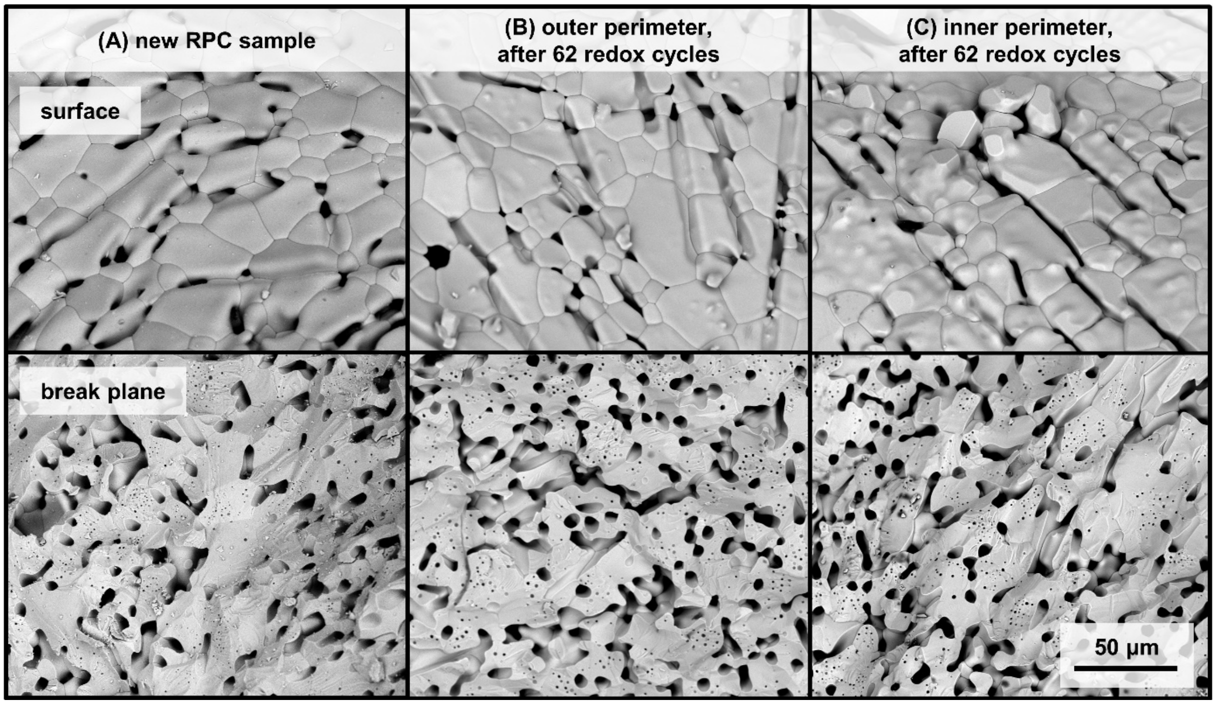

Figure S7. SEM images of the RPC strut surfaces (top row) and break plane cross sections (bottom row) of three different samples extracted from: (A) an unreacted RPC; (B) the outer perimeter of the front lateral ring of RPC bricks (facing the reactor insulation) after 62 cycles; and (C) the directly irradiated inner perimeter of the front lateral ring of RPC bricks after 62 cycles. All images are on the same scale.

Gas-to-Liquid Unit

As shown in Figure 1, the GtL conversion subsystem unit sits at the base of the solar tower. The cobalt-based catalyst system was designed by the industrial partner HyGear. The primary components of the unit, all housed and integrated into a modular container, are indicated in Figure S10. By inclusion of the buffer tank, the GtL reactor could be operated to meet any desired production schedule. The syngas composition in the buffer tank is estimated in real time by integration of gas analysis and flow rate data from the solar reactor subsystem and verified directly by sampling the tank for subsequent gas chromatography. The GtL controller automatically draws syngas from the pressurized buffer tank to perform the catalytic FT synthesis at 30 bar and 210 °C. The Co-based catalyst requires an H_2:CO molar ratio of around 2.15,[50,51] which the solar reactor subsystem is able to match very closely. The outflow stream is decompressed and cooled in stages to ambient temperature to separate the wax and liquid products from the uncondensed stream which contains unreacted syngas and gaseous hydrocarbon species C1-C4. To increase the chemical conversion to long-chain hydrocarbons, this gaseous mixture is recycled by reforming it to H_2 and CO, and finally blending it with the solar syngas coming from the solar reactor. The reformer is heated electrically, but in principle it could be driven by waste heat from the solar reactor. Figure S8 shows the concentration of carbon species in both the liquid hydrocarbon fraction and the wax, analyzed by GC/MS. The measured hydrocarbons comprise aliphatic species. The liquid fraction contained a mixture of 16% kerosene (C10-C15), 40% diesel (C15-C20), and C20+ hydrocarbons up to C34. The wax contained 7% kerosene, 40% diesel, and C20+ molecules up to C43.

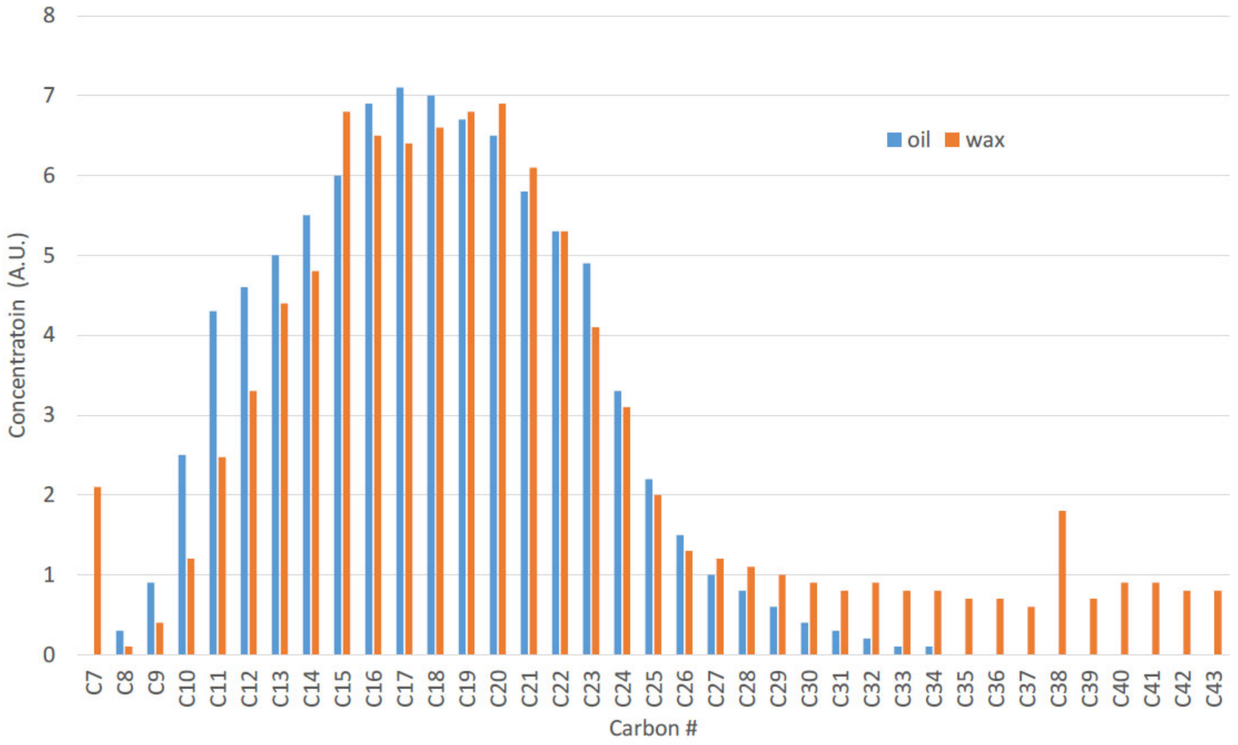

Figure S8. GC/MS analysis of the liquid fraction and wax phase of the products of the FT synthesis, produced from solar syngas in the GtL unit.

Solar Reactor

The 50 kW solar reactor was introduced previously as part of a heat transfer modelling study[52] and briefly described in a publication presenting the EU project SUN-to-LIQUID.[49] It is described in significant detail here. Development of the solar reactor evolved from an original design utilizing monolithic ceria,[53] to its present configuration utilizing a reticulated porous ceramic (RPC) structure.[42] The reactors configuration is schematically shown in Figure 4. It consists of a cavity-receiver with a 160 cm diameter circular aperture through which concentrated solar radiation enters. The aperture is sealed with a 12 mm-thick, 300 mm diameter. quartz disk window, which is mounted on a water-cooled aluminum front shield fixed to the reactor vessel. The window is actively cooled with a continuous air flow directed onto its outer surface via four nozzle jets. The reactor vessel is made of stainless steel and is pressure tight for operation under vacuum. It is internally lined with Al_2O_3−SiO_2 thermal insulation (Rath, Inc., type KVS 184/400) and externally covered by a detachable insulating jacket made from woven glass fibers and filled with ceramic mat board. The inner walls of the cavity consist of an interlocking structure of RPC bricks, made

of pure ceria, approaching the shape of a cylinder closed at one end. Reactant CO_2 and inert Ar gases enter the reactor via tangential inlet ports located behind the window and oriented to form a vortex flow which cools and protects the window from dust deposition. H_2O is introduced via a separate inlet through the front-side thermal insulation. Product gases exit the solar reactor axially via an outlet port at the rear of the reactor vessel. The solar reactor is mounted on top of the solar tower and tilted downwards towards the heliostat field with an inclination angle of 40 degrees. To prevent structural failure of the RPC cavity in this orientation, the RPC bricks are arranged in a self-supporting layout, shown in detail in Figure S9. The side of the cavity consists of two rings assembled out of 16 separate RPC bricks each. The dome-shaped back design consists of eight wedge-shaped pieces that are inclined by 30 degrees relative to a flat back plane, and one central octagonal piece with chamfered edges, acting as the keystone for the interlocking geometry. While in the down-facing operating angle, the design keeps all of the back pieces in compression against each other, just as the interlocking rings of side bricks hold each other in compression. The total ceria mass of all RPC bricks forming the cavity is between 18.1 kg and 21.3 kg, with each RPC brick having a thickness of 35 mm and a pore size of approximately 7 ppi (pores per inch). The bricks were manufactured using the Schwartzwalder replication method with dual-scale porosity: millimeter-scale pores made from struts containing micrometer-scale pores (Figure S9).[54,55] The millimeter-scale pores enhance the volumetric absorption of concentrated solar radiation during the reduction step, while the micrometer-scale pores within the struts enhance the reaction kinetics during the oxidation step.[55]

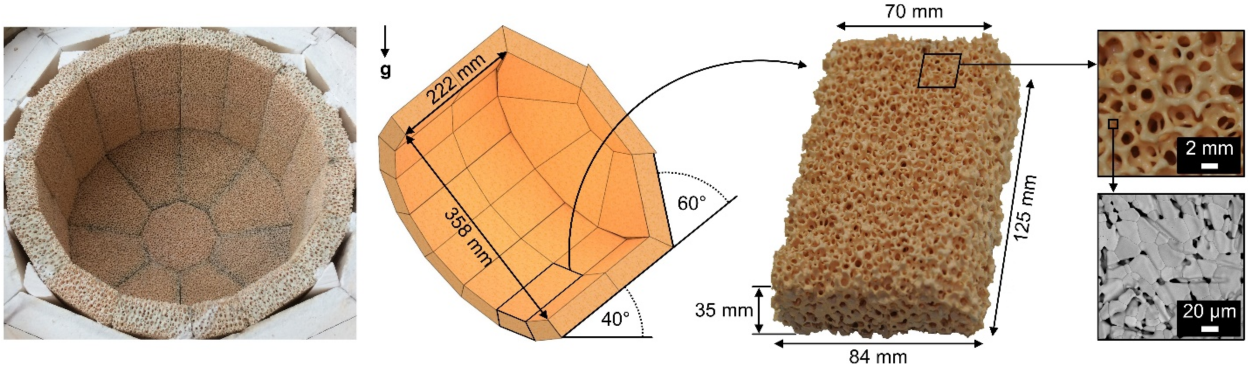

Figure S9: Details on the self-supporting, interlocking ceria structure assembled out of 41 separate RPC bricks, including the center-back keystone. The RPC features dual-scale porosity: millimeter-scale pores made by struts which contain micrometer-scale pores.

Experimental Setup

The experimental setup installed in the solar tower and the adjacent GtL unit is schematically depicted in Figure S10. Gas flow rates of Ar and CO_2 are regulated using electronic mass flow controllers (Bronkhorst, EL-FLOW Select). Liquid water is fed with a stepper motor-driven positive displacement pump into an electrically-heated steam generator (Adrop Feuchtemesstechnik GmbH, ATHMOS-RS-4). The steam is superheated to 260 °C and fed into the reactor cavity through the thermal insulation via an electrically heated Teflon pipe, entering the cavity at roughly 200 °C. The enthalpy of water vaporization is assumed to be supplied by either waste heat from the solar reactor, spilled radiation from the solar concentrating system, or the exothermic GtL unit, or by Q_{solar} if liquid water is fed directly to the solar reactor. For convenience, external steam generation was used in our setup and its energy penalty, which for reference represents less than 0.5% of the solar radiative power delivered to the tower from the heliostat field, was neglected. The temperature of the reacting ceria is monitored at four positions distributed over the back surface of the RPC using B-type thermocouples. The average of these temperature measurements is defined as the nominal RPC temperature; all RPC temperatures reported refer to this mean value. The pressure inside the reactor is measured at the gas outlet and through a lateral port using Pirani gauge sensors combined with capacitance diaphragm vacuum gauges (Leybold, THERMOVAC, TTR 101 N). The reported reactor pressure corresponds to the average of both pressure measurements. Up to three dry, multi-stage root vacuum pumps (Pfeiffer Vacuum, ACP 40) are attached in parallel downstream of the outlet port of the solar reactor using a 50 mm-dia. stainless steel flexible vacuum hose. A solenoid control valve (Bürkert Schweiz AG, valve type 2875, controller type 8605) is used to slowly evacuate the reactor at the beginning of the reduction step (path shown by the red lines in Figure S10), and an electro-pneumatic valve with bigger nominal diameter (SMC Corporation, XLAV-50) is opened once the pressure was below 200 mbar. During the oxidation step (path shown by the blue lines in Figure S10), the vacuum pumps are bypassed by use of a smaller diameter electro-pneumatic valve (SMC Corporation, EVNB211B). Unreacted water is removed with a water-cooled, standard laboratory condenser (Dimroth type, made from DURAN® glass). Product gas composition is continuously analyzed downstream via a parallel diversion of sample gas using a paramagnetic sensor for O_2 (Siemens, Ultramat 23, frequency 1 Hz), IR detectors for CO and CO_2 (Siemens, Ultramat 23, frequency 1 Hz), and a thermal conductivity-based detector for H_2 (Siemens, Calomat 6, frequency 1 Hz).

Gas chromatography (Agilent, 490 Micro GC, frequency 0.012 Hz) is used for verification and for estimating the accuracy of the final gas composition.

During the endothermic reduction step, Ar flow is used to protect the quartz window and is pumped out together with the released O_2 while the solar reactor is under vacuum and heated with concentrated solar radiation. During the exothermic oxidation step, the input of solar radiation is interrupted and the ceria is re-oxidized with a mixture of H_2O and CO_2 under atmospheric pressure, co-producing H_2 and CO (syngas). The O_2 released during the reduction step is vented together with the inert Ar gas flow. The syngas produced during the oxidation step is compressed to 30–150 bar and stored intermediately in a 50 L gas cylinder to be subsequently processed in the GtL unit at the base of the solar tower.

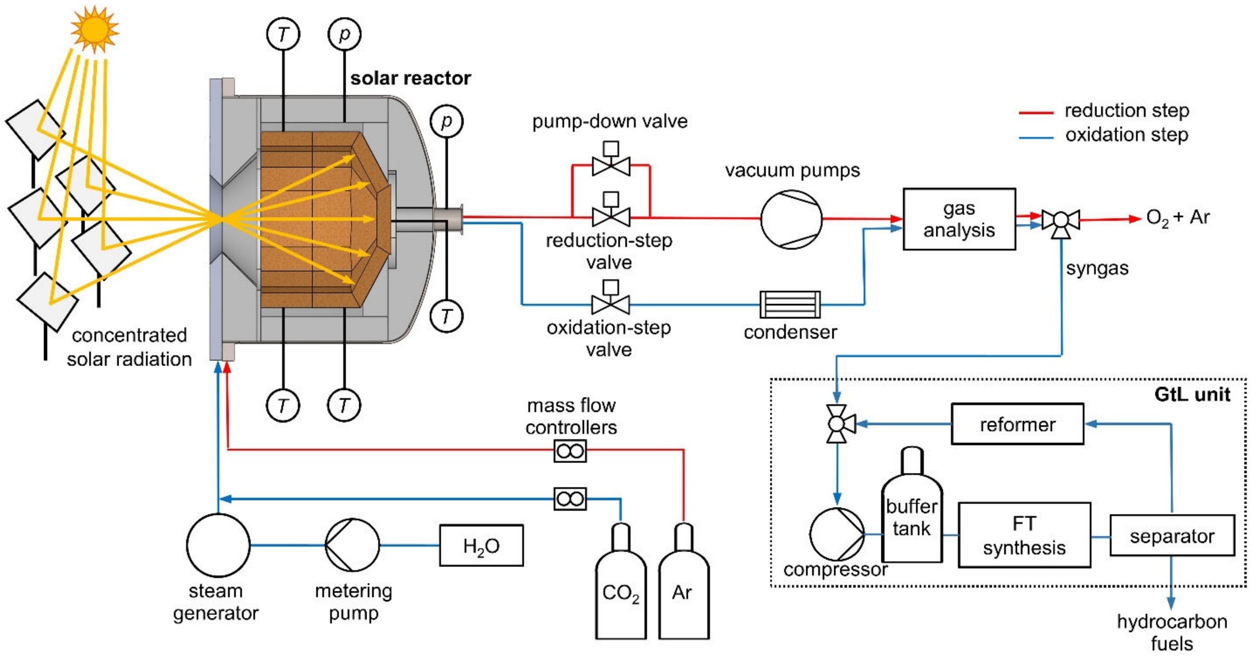

Figure S10. Simplified schematic of the experimental setup, comprising three fully-integrated subsystems: (1) the solar tower concentrating facility, (2) the solar reactor with its peripheral components, and (3) the gas-to-liquid (GtL) unit. *Red line*: flow path during the reduction step. *Blue line*: flow path during the oxidation step.

Nomenclature

E_{inert}	energy required for inert gas separation (J mol^{-1})
ΔH_{CO}	heating value of CO (J mol^{-1})
ΔH_{H_2}	higher heating value of H_2 (J mol^{-1})
m_{RPC}	ceria RPC cavity mass loading (kg)
$\dot{n}$	molar gas flow rate (mol s^{-1})
$\dot{n}_{CO_2}$	molar flow rate of CO_2 (mol s^{-1})
$\dot{n}_{H_2O}$	molar flow rate of H_2O (mol s^{-1})
p_{atm}	atmospheric pressure (Pa)
p	pressure (Pa)
P_{solar}	solar radiative power input (kW)
Q_{syngas}	heating value of syngas produced (J)
Q_{inert}	heat equivalent of work for inert gas separation (J)
Q_{input}	total thermal energy input ($Q_{input} = Q_{solar} + Q_{pump} + Q_{inert}$) (J)
Q_{pump}	heat equivalent of work for vacuum pumping (J)
Q_{solar}	solar radiative energy input (J)
r_{CO}	molar rate of CO production (mol s^{-1})
r_{H_2}	molar rate of H_2 production (mol s^{-1})
r_{inert}	inert gas flow rate (mol s^{-1})
R	universal gas constant (8.314 J K^{-1} mol^{-1})
t	time (s)
T_{pump}	pump temperature (298.15 K)
$\dot{V}_{Ar}$	volumetric flow rate of Argon (L min^{-1})
δ	nonstoichiometry of ceria
η_{GtL}	gas-to-liquid energy efficiency
$\eta_{heat\text{-}to\text{-}work}$	heat-to-work energy efficiency
$\eta_{optical}$	optical efficiency of the solar concentrating sub-system
η_{pump}	vacuum pump efficiency
$\eta_{solar\text{-}to\text{-}syngas}$	solar-to-syngas energy efficiency

Abbreviations

CCD	charge-coupled device
CFD	computational fluid dynamics

DNI	direct normal irradiance
FMAS	flux measurement acquisition system
FT	Fischer-Tropsch
GC	gas chromatography
GtL	gas-to-liquid
MS	mass spectrometry
ppi	pores per inch
RPC	reticulated porous ceramic
SEM	scanning electron microscope

Supplemental References

41. Romero, M., González-Aguilar, J., and Luque, S. (2017). Ultra-modular 500m^2 heliostat field for high flux/high temperature solar-driven processes. In *AIP Conference Proceedings*. 1850 (1). AIP Publishing. 10.1063/1.4984387.

42. Marxer, D., Furler, P., Takacs, M., and Steinfeld, A. (2017). Solar thermochemical splitting of CO_2 into separate streams of CO and O_2 with high selectivity, stability, conversion, and efficiency. Energy & Environmental Science *10*, 1142-1149. 10.1039/c6ee03776c.

43. Bulfin, B., Miranda, M., and Steinfeld, A. (2021). Performance indicators for benchmarking solar thermochemical fuel processes and reactors. Frontiers in Energy Research *9*. 10.3389/fenrg.2021.677980.

44. Ermanoski, I., Siegel, N.P., and Stechel, E.B. (2013). A new reactor concept for efficient solar-thermochemical fuel production. Journal of Solar Energy Engineering *135*, 031002. 10.1115/1.4023356.

45. Brendelberger, S., and Sattler, C. (2015). Concept analysis of an indirect particle-based redox process for solar-driven H_2O/CO_2 splitting. Solar Energy *113*, 158-170. 10.1016/j.solener.2014.12.035.

46. Häring, H. (2008). The Air Gases Nitrogen, Oxygen and Argon. In Industrial Gases Processing, (Wiley-VCH Verlag GmbH & Co. KGaA), pp. 9-109. 10.1002/9783527621248.ch2.

47. Schubnell, M., Keller, J., and Imhof, A. (1991). Flux density distribution in the focal region of a solar concentrator system. Journal of Solar Energy Engineering *113*, 112–116. 10.1115/1.2929954.

48. Thelen, M., Raeder, C., Willsch, C., and Dibowski, G. (2017). A high-resolution optical measurement system for rapid acquisition of radiation flux density maps. In *AIP Conference Proceedings*. 1850 (1). AIP Publishing. 10.1063/1.4984534.

49. Koepf, E., Zoller, S., Luque, S., Thelen, M., Brendelberger, S., González-Aguilar, J., Romero, M., and Steinfeld, A. (2019). Liquid fuels from concentrated sunlight: An overview on development and integration of a 50 kW solar thermochemical reactor and

high concentration solar field for the SUN-to-LIQUID project. In *AIP Conference Proceedings*. 2126 (1). AIP Publishing. 10.1063/1.5117692.

50. Dry, M.E. (2002). The Fischer–Tropsch process: 1950–2000. Catalysis today *71*, 227-241. 10.1016/S0920-5861(01)00453-9.

51. Iglesia, E. (1997). Design, synthesis, and use of cobalt-based Fischer-Tropsch synthesis catalysts. Applied Catalysis A: General *161*, 59-78. 10.1016/S0926-860X(97)00186-5.

52. Zoller, S., Koepf, E., Roos, P., and Steinfeld, A. (2019). Heat Transfer Model of a 50 kW Solar Receiver–Reactor for Thermochemical Redox Cycling Using Cerium Dioxide. Journal of Solar Energy Engineering *141*, 021014. 10.1115/1.4042059.

53. Chueh, W.C., Falter, C., Abbott, M., Scipio, D., Furler, P., Haile, S.M., and Steinfeld, A. (2010). High-Flux Solar-Driven Thermochemical Dissociation of CO_2 and H_2O Using Nonstoichiometric Ceria. Science *330*, 1797-1801. 10.1126/science.1197834.

54. Furler, P., Scheffe, J., Gorbar, M., Moes, L., Vogt, U., and Steinfeld, A. (2012). Solar thermochemical CO_2 splitting utilizing a reticulated porous ceria redox system. Energy & Fuels *26*, 7051-7059. 10.1021/ef3013757.

55. Furler, P., Scheffe, J., Marxer, D., Gorbar, M., Bonk, A., Vogt, U., and Steinfeld, A. (2014). Thermochemical CO_2 splitting via redox cycling of ceria reticulated foam structures with dual-scale porosities. Physical Chemistry Chemical Physics *16*, 10503-10511. 10.1039/C4CP01172D.
